# FedPSFV: Personalized Federated Learning via Prototype Sharing for Finger Vein Recognition

**DOI:** 10.3390/s25216790

**Published:** 2025-11-06

**Authors:** Haoyan Xu, Yuyang Guo, Yunzan Qu, Jian Guo, Hengyi Ren

**Affiliations:** 1 School of Computer Science, Nanjing University of Posts and Telecommunications, Nanjing 210023, China; b22140827@njupt.edu.cn (H.X.);; 2 Jiangsu High Technology Research Key Laboratory for Wireless Sensor Networks, Nanjing University of Posts and Telecommunications, Nanjing 210023, China; 3 College of Information Science and Technology & Artificial Intelligence, Nanjing Forestry University, Nanjing 210037, China

**Keywords:** finger vein recognition, federated learning, margin-based loss function, prototype sharing

## Abstract

Finger vein recognition algorithms based on deep learning techniques are widely used in many fields. However, the training of finger vein recognition models is hindered by privacy issues and the scarcity of public datasets. Although applying federated learning techniques to finger vein recognition can effectively address privacy concerns, data heterogeneity across clients limits the performance of the models, especially on small datasets. To address these problems, in this paper, we propose a new federated finger vein recognition algorithm (FedPSFV). The algorithm is based on the federated learning framework, which increases the interclass distance of each dataset by sharing the prototypes among clients to solve the data heterogeneity problem. The algorithm also integrates and improves the margin-based loss function, which advances the feature differentiation ability of the model. Comparative experiments based on six public datasets (SDUMLA, MMCBNU, USM, UTFVP, VERA, and NUPT) show that FedPSFV has better accuracy and generalizability; the TAR@FAR = 0.01 is improved by 5.00–11.25%, and the EER is reduced by 81.48–90.22% compared to the existing methods.

## 1. Introduction

Finger vein recognition is a biometric technology that authenticates individuals by detecting vein patterns within a finger. In contrast to other recognition technologies, such as facial recognition and fingerprint recognition, finger vein recognition relies on blood flow imaging to enable liveness detection. This feature makes counterfeiting and stealing more difficult and renders the technology more suitable for locations with high security requirements.

Recently, deep learning techniques based on neural networks have become the mainstream methods for finger vein recognition [1,2,3,4]; these methods can automatically learn features and are highly scalable. Neural network training usually requires large datasets [5]. However, owing to legal restrictions such as privacy protection, research institutes and related organizations face many limitations in collecting and sharing finger vein data [6,7,8]. As a result, these organizations are only able to train models on a limited number of local datasets, resulting in low recognition accuracies of the obtained models.

Federated learning (FL), which allows participating clients to jointly train models without sharing datasets and only exchanges training parameters, is a potential solution to data privacy issues [9,10,11,12]. Currently, federated learning techniques have been applied in the fields of facial recognition and iris recognition [13,14,15,16,17] with good results, and some methods, such as FedFV [18], DDP-FedFV [19], and FVRFL [20], have been proposed in the field of finger vein recognition. These methods avoid privacy risks. However, due to differences in the acquisition methods, external environment, users, imaging quality, number of images, etc., the data from each client are extremely varied, which results in poor generalizability of the models trained by the existing methods. Performance degradation is particularly obvious for some clients with small data volumes [19].

To address the above limitations, this paper presents an algorithm named FedPSFV. FedPSFV addresses the data heterogeneity problem by sharing prototypes among different clients, which increases the generalizability of the model. Moreover, FedPSFV integrates a margin-based loss function to further increase the model’s feature differentiation ability. The main research contributions of this paper are as follows.

(1) A novel federated finger vein recognition algorithm named FedPSFV is proposed. FedPSFV applies the concept of prototype sharing, which addresses the client data heterogeneity problem in federated learning. By sharing the prototype, the recognition performance of each client is improved.

(2) The margin-based loss function is presented and improved in the federated learning framework. This framework enables the margin-based loss function to adjust the spacing between samples more accurately on the basis of the edge intensity and increases the feature differentiation ability of the model.

(3) Open-set tests on six public heterogeneous finger vein datasets (SDUMLA, MMCBNU, USM, UTFVP, VERA, and NUPT) reveal that the FedPSFV algorithm is effective in addressing the data heterogeneity problem and outperforms the recognition performance of the existing classical methods.

The following sections are organized as follows. Section 2 reviews related work in the fields of finger vein recognition and federated learning. Section 3 presents the detailed implementation of FedPSFV. Section 4 discusses the performance of FedPSFV on the basis of the experimental results. Section 5 presents the conclusion and discusses the limitations of FedPSFV and potential directions for future work.

## 2. Related Work

### 2.1. Finger Vein Recognition

Finger vein recognition is an emerging biometric identification technology, and the main process has four stages: image acquisition, image preprocessing, feature extraction, and matching recognition. Feature extraction is the most critical step and affects the recognition accuracy of the entire system directly. Classical manual feature extraction algorithms include the repetitive line tracking (RLT) algorithm, the local maximum curvature (LMC) algorithm, the principal curvature (PC) algorithm, and the local binary pattern (LBP) method [21,22,23]. Overall, these methods have average recognition accuracy and poor scalability.

Recently, an increasing number of studies have applied deep learning algorithms to finger vein recognition. Feature extraction algorithms based on deep learning not only improve recognition performance but also simplify data processing operations. Hu et al. [24] trained a CNN containing 10 convolutional layers for finger vein recognition and achieved good results. Zhao et al. [25] combined center loss and Softmax loss and increased the accuracy and speed of finger vein recognition via dynamic regularization, which outperformed the classical deep learning algorithms on the MMCBNU_6000 and FV-USM datasets. Song et al. [26] used composite images to train a modified DenseNet, which increased the feature extraction capability of the model. These methods can often achieve better results than traditional methods; however, they usually require large datasets to train the neural network [19]. It is often difficult for research institutions to obtain and share large finger vein datasets because of privacy protection, data security, and other limitations, which makes training models with high generalizability challenging.

### 2.2. Finger Vein Recognition via Federated Learning

Federated learning is a distributed machine learning framework that enables multiple participants to jointly train models without the sharing of local data. This framework protects the privacy of customer data and satisfies the requirements of the biometrics domain. Currently, federated learning-related algorithms have been continuously proposed [27,28,29,30] and are gradually being used in facial recognition, iris recognition, and other fields [31]. In the field of finger vein recognition, much related research is still in its infancy. FedFV [18] proposed an innovative parameter aggregation method that reduces the computational cost of the personalized aggregation method. DDP-FedFV [19] proposed a two-phase federated learning algorithm for the field of finger vein recognition. However, multiparty collaborative training also introduces a new problem: different clients have data with different data qualities and different data sizes. The existing federated learning frameworks have difficulty addressing these differences, and the trained models are not sufficiently discriminative of features and have large performance fluctuations on different client datasets.

To address these limitations, this paper increases the adaptability of the model to different datasets by sharing prototypes among different clients. Moreover, an improved margin-based loss function that improves the feature discrimination ability of the model is presented.

## 3. Methodology

This section describes the problem of the FL-based finger vein recognition algorithm, presents the overall framework of the FedPSFV algorithm, and presents the specific implementation of each module of the algorithm.

### 3.1. Problem Description

The method in this paper uses the federated learning framework. Assuming a total of *m* different clients, each client is denoted as *C_k_* (*k =* 1, 2*…m*), and the total number of categories in the dataset of all clients is *N*. For the *k*-th client, its local dataset is denoted as *D_k_* ∈ *X_k_* × *Y_k_*. *X_k_* and *Y_k_* denote the feature space and label space, respectively, of the dataset of the *k*-th client.

In the FedPSFV algorithm, the model $F\left(\omega ;x\right)$ with the same architecture is shared among clients, where *ω* denotes the learnable weights of the model and *x* denotes the input features. The objective function of the FedPSFV algorithm is defined in Equation (1),

(1) $$\underset{\omega }{min}\sum_{k\;=\;1}^{m}\frac{{|D}_{k}|}{N}L_{FedPSFV}\left(F\left(\omega ;x\right),y\right)$$

where *y* denotes the corresponding labeled value and $L_{FedPSFV}$ denotes the loss function.

### 3.2. Framework of the FedPSFV Method

**Figure 1:**
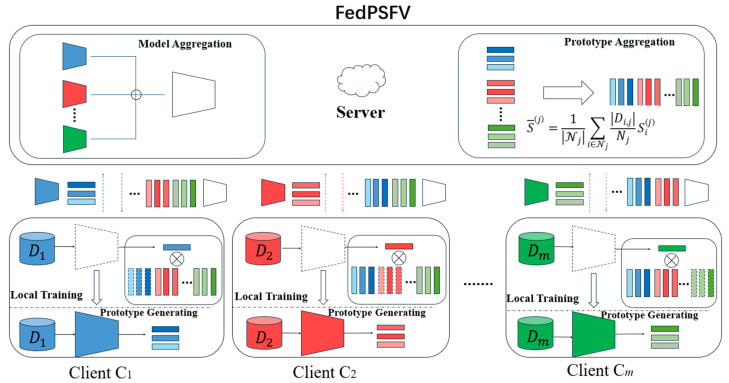
Framework of the FedPSFV algorithm, which contains *m* clients. The trapezoidal shape represents the feature representation layer of the model, and the horizontal rectangle represents the prototype. The vertical dashed rectangle represents the classifier weight matrix obtained from the local initialization of the clients, and the vertical solid rectangle represents the expanded classifier weight matrix. After completing a training epoch, each client generates prototypes and uploads them. The server then aggregates the prototypes and model parameters and sends the updated information back to the clients.

This section briefly describes the framework of the FedPSFV method. The overall framework of the algorithm, which is shown in Figure 1, contains two parts: the client side and the server side. The client side completes the preprocessing of the finger vein data before training, and then during each communication round of federated learning, after the client has trained the local model, it computes the prototypes and uploads them and the model parameters to the server side. The server side receives and aggregates all the uploaded prototypes and model parameters and then sends the prototypes and the model parameters down to each client to participate in the next round of training.

The specific implementation details of the FedPSFV algorithm are described in Section 3.3 and Section 3.4. Section 3.3 describes the client-side training framework, which contains the data preprocessing, classification layer, and loss function of the FedPSFV algorithm, and Section 3.4 describes how to aggregate the model parameters and the prototypes on the server side.

### 3.3. Local Training Process

**Figure 2:**
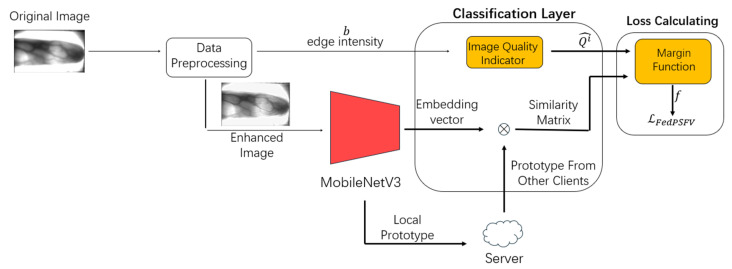
Local training process for the client. The data preprocessing module performs image enhancement and edge intensity calculations on the finger vein samples. The edge intensity is used as a quality evaluation metric to calculate the quality score of the image. The enhanced image enters the feature extraction layer to obtain the embedding vector and then enters the classification layer, which is multiplied by the classifier weight matrix composed of the prototype to obtain the vector angle and similarity matrix. Finally, the loss function is computed using an image quality indicator and the vector angle.

This section describes the training process of the local client, which includes data preprocessing, network training, prototype calculation, and loss calculation. Figure 2 shows the training process of the local client.

The next section describes the various client modules in detail.

#### 3.3.1. Data Preprocessing

**Figure 3:**
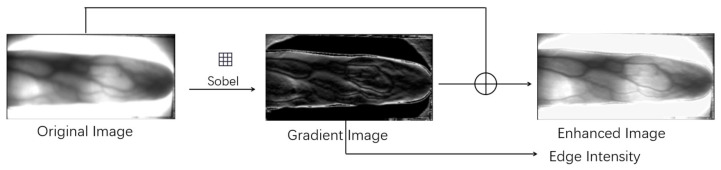
Preprocessing of finger vein samples. The gradient image is obtained by applying the Sobel operator to the original image. The enhanced image is then generated by adding the original image and the gradient image. The gradient image is also used to compute the edge intensity.

The FedPSFV algorithm has two main preprocessing tasks. The first task is image enhancement, which highlights the key details of the finger veins and facilitates the extraction of discriminative features by the subsequent model. The second task is to calculate the edge intensity for the subsequent image quality indicator. The main preprocessing process is shown in Figure 3.

When the image enhancement process is performed on the client side, the gradient image *I_g_* of the finger vein image *I* is calculated and then added to the original image to obtain the enhanced image $I^{\prime }$. The process is shown in Equation (2),

(2) $$I^{\prime }\;=\;I\;+\;I_{g}$$

where the gradient image is computed via the Sobel operator, as expressed in Equation (3).

(3) $$I_{g}\;=\;\left\{\begin{array}{c} 0,\;\;if\;|G\;\times \;I|\;>\;T\\ |G\;\times \;I|,\;\;if\;|G\;\times \;I|\;\leq \;T \end{array}\right.$$

Here, *T* represents the gradient threshold, which is usually 100, and *G* represents the Sobel operator. When the finger orientation of the finger vein image is vertical, the value of *G* is as shown in Equation (4). When the finger orientation is horizontal, *G* is transposed and substituted into Equation (3) for calculation.

(4) $$G\;=\;\left(\begin{array}{ccc} -1 & 0 & 1\\ -2 & 0 & 2\\ -1 & 0 & 1 \end{array}\right)$$

While the image is enhanced, the client will synchronize the calculation of the edge intensity *b* of the finger vein. The process is shown in Equation (5),

(5) $$b\;=\;\sum_{px,py}I_{g}\left(px,py\right)$$

where *I_g_*(*px*, *py*) denotes the pixel value with coordinates (*px*, *py*) in the gradient image.

For the same category, the client calculates the edge intensity of all the samples of the category and calculates the average value as the edge intensity of the category.

#### 3.3.2. Local Network Architecture

The local neural network model of the client consists of two parts: a feature extraction layer and a classification layer. The feature extraction layer of the FedPSFV algorithm uses the MobileNetV3 network [32], and the classification layer uses the designed EdgeAdavein module. Each of these layers is described below.

The preprocessed images enter the feature extraction layer, which is the MobileNetV3 network. The feature extraction layer needs to calculate the local prototype after each round of training and upload it to the server; additionally, it needs to output the embedding vector to the classification layer during training for the calculation of the loss function.

The calculation process of the prototype is presented. A prototype is the center of a feature representation of a class and is usually expressed as the mean of the embedding vectors. Assume that client *k* has a dataset *D_k_* of *N_k_* categories and that *D_k_* = $\left\{D_{k}^{1},D_{k}^{2},\ldots \ldots D_{k}^{N_{k}}\right\}$, where $D_{k}^{j}$ denotes the set of all training samples of category *j* on client *k* and $S_{k}^{j}$ represents its corresponding prototype, which is calculated as shown in (6),

(6) $$S_{k}^{j}\;=\;\frac{1}{\left|D_{k}^{j}\right|}\sum_{\left(x,y\right)\in D_{k}^{j}}f_{k}\left(x\right)$$

where *f_k_*(*x*) denotes the embedding vector obtained after the input feature *x* passes through the feature extraction layer and $\left|D_{k}^{j}\right|$ denotes the number of training samples of category *j*. At the end of each training batch, the client inputs the image data into the feature extraction layer to obtain the embedding vector and computes the local prototype via Equation (6), which is then uploaded to the server side with the model parameters.

After the feature extraction layer is processed, the embedding vectors of its output are fed into the classification layer. The FedPSFV algorithm, which designs the EdgeAdavein module as the classification layer, has two main tasks: (1) calculating the similarity matrix and (2) calculating the image quality indicator using the edge intensity.

The similarity matrix is constructed. The similarity matrix represents the degree of similarity between any two objects in the dataset, where each column vector ${vc}_{j}$ represents the degree of similarity between all the samples of the current batch and the representative vector of category *j*. The similarity matrix is obtained by multiplying the normalized embedding vector by the classifier weight matrix. In the classification layer, the similarity matrix is obtained by multiplying the embedding vector by the classifier weight matrix.

A classifier weight matrix is a parameter matrix used in machine learning to map input features into category predictions and to represent the centers of categories [33,34]. In federated learning, the traditional classifier weight matrix represents only the centers of all the categories in the local dataset. This approach results in a similarity matrix that represents only the similarity between the current batch of data and all the local data but not the similarity between the current batch of data and all the client data, which affects the feature differentiation ability of the model. To address this limitation, the FedPSFV algorithm expands the local classifier weight matrix of the client to include information about the data of other clients. However, instead of simply using the classifier weight matrices of other clients to expand the local classifier weight matrix, FedPSFV uses the prototypes of the other clients. The reasons and benefits of this treatment are as follows.

(1) In practice, the embedding vectors obtained from different categories of samples through the embedding function, not the weight vectors of the weight matrix, must be distinguished. The prototype is the average of the embedding vectors of all the samples of a category, so the prototype more effectively enhances the feature discrimination capability of the model.

(2) The weight vector of the classifier weight matrix, although it also considered the center of the category, is randomly initialized and updated by backpropagation several times at the end of each round of training, which is highly affected by the hyperparameters of the optimizer, and the values are not sufficiently stable in comparison to those of the prototype.

Next, we discuss ways to expand the local classifier weight matrix with prototypes in the EdgeAdavein module. Assume that there are *m* clients and that the local classifier weight matrix of the *k*-th client is *W_k_*, as shown in Equation (7). The prototype matrix of other clients *a* is *P_a_*, as shown in Equation (8),

(7) $$W_{k}\;=\;\left(\begin{array}{ccc} w_{1,1} & \ldots & w_{1,N_{k}}\\ \vdots & \ddots & \vdots \\ w_{d,1} & \cdots & w_{d,N_{k}} \end{array}\right)$$

(8) $$P_{a}\;=\;\left(\begin{array}{ccc} S_{a}^{1} & \cdots & S_{a}^{N_{a}} \end{array}\right)$$

where $w_{v,z}$ denotes the local classifier weight of client *k*, and 1 ≤ *v ≤ d*, 1 ≤ *z ≤ N_k_*; $S_{a}^{j}$ denotes the prototype of category *j* in client *a* sent by the server, which is a vector of *d* dimensions, and 1 ≤ *a ≤ m*, 1 ≤ *j ≤ N_a_*; *N_k_* and *N_a_* denote the total number of categories for clients *k* and *a*, respectively; and *d* represents the number of output dimensions of the feature extraction layer MobileNetV3.

The expanded local classifier weight matrix ${W\prime }_{k}$ for client *k* is shown in Equation (9).

(9) $${W\prime }_{k}\;=\;\begin{array}{ccc} {(P}_{1} & P_{2} & \cdots \end{array}\begin{array}{ccc} P_{k\;-\;1} & W_{k} & P_{k\;+\;1} \end{array}\cdots \begin{array}{cc} P_{m\;-\;1} & P_{m} \end{array})$$

The similarity matrix of the FedPSFV algorithm is obtained by multiplying all the embedding vectors of a batch by the expanded classifier weight matrix.

The next section describes the computation of the image quality indicator in the EdgeAdavein module, which consists of two main parts: normalization of the edge intensity and a quality score computation. Assume that the set of edge intensities *B_k_* = $\left\{b_{k}^{1},b_{k}^{2},\ldots ,b_{k}^{i},\ldots b_{k}^{N_{k}}\right\}$ for the dataset of client *k* and that $b_{k}^{i}$ is the edge intensity of category *i*. Then, for category *i*, its quality fraction *Q^i^* is calculated as shown in Equations (10) and (11),

(10) $${b^{\prime }}_{k}^{i}\;=\;\frac{b_{k}^{i}\;-\;\min\left(B_{k}\right)}{max\left(B_{k}\right)\;-\;min\left(B_{k}\right)}$$

(11) $$Q^{i}\;=\;\frac{100}{1\;+\;e^{-u\cdot \left({b^{\prime }}_{k}^{i}\;-\;0.5\right)}}$$

where ${b^{\prime }}_{k}^{i}$ denotes the normalized edge intensity; the *min* and *max* functions denote the minimum and maximum values, respectively, taken over the set; and *u* represents a parameter that controls the steepness of the function curve, which is typically 10. The above processing converts the edge intensities to a quality score ranging from 0 to 100. The quality fraction is subsequently normalized to $\hat{Q^{i}}$, as shown in Equation (12), and it is used as an image quality indicator to calculate the margin loss.

(12) $$\hat{Q^{i}}\;=\;{\left[\frac{Q^{i}-\mu_{Q}}{\sigma_{Q}}\cdot h\right]}_{-1}^{1}$$

Here, $\mu_{Q}$ and $\sigma_{Q}$ represent the mean and standard deviation, respectively, of all $Q^{i}$ s within a batch; ${\left[⬚\right]}_{-1}^{1}$ indicates that the value is intercepted in the range of −1 to 1 to increase numerical stability; and *h* is a hyperparameter that is typically 0.33.

#### 3.3.3. Loss Calculation

The loss function is often used in deep learning to quantify the difference between the prediction result of the model and the true label value, and the setting of the loss function often determines the feature discrimination ability of the model. Traditional loss functions, such as the Softmax loss function, focus only on maximizing the probability of the correct class and do not sufficiently consider the distance within the class, resulting in insufficient feature discrimination. For this reason, this paper presents the margin-based loss function [35,36,37], which not only reduces the intraclass distance of the local dataset but also increases the interclass distance from other client datasets in combination with the shared prototype.

The margin-based loss function $f{\left(\theta_{j},m\right)}_{Edge}$ designed in this paper is shown in Equation (13),

(13) $$f{\left(\theta_{j},m\right)}_{Edge}\;=\;\left\{\begin{array}{c} s(\cos\left(\theta_{j}\;+\;g_{angle}\right)\;-\;g_{add})\;j\;=\;y_{i}\\ s\cos\theta_{j}\;\;\;\;\;\;\;\;\;j\;\neq \;y_{i} \end{array}\right.$$

where *s* represents the hyperparameter scaling factor, $\theta_{j}$ denotes the value of Row *i* and Column *j* of the similarity matrix, representing the angle between the embedding vector of category *i* and the column vector of category *j* in the expanded classifier weight matrix; *y_i_* denotes the labeled value of category *i*; and *g_angle_* and *g_add_* denote the additive margins in angular space and cosine space, respectively, with the formulas shown in (14) and (15),

(14) $$g_{\mathrm{angle}}\;=\;-m\cdot \hat{Q^{i}}$$

(15) $$g_{\mathrm{add}}\;=\;m\cdot \hat{Q^{i}}\;+\;m$$

where *m* represents the margin, a type of hyperparameter, which is typically 0.4.

The pseudocode that calculates edge intensity and margin loss is shown in Algorithm 1.
**Algorithm 1.** Calculating edge intensity and margin loss**Parameters**: dataset of client *D_k_*, sample set $d_{k}^{a}\in D_{k}$ belonging to a class of *a*, sample image *I* belongs to a sample set, edge intensity set *B_k_* of the client, and quality score set *Q_k_* of the client. Every client executes:1:Initialize the local classifier weight matrix *W_k_* and hyperparameter margin *m*2:**for** $\;d_{k}^{i}\;=\;d_{k}^{1},d_{k}^{2},\ldots \ldots d_{k}^{N_{k}}$** do**3:  **set** the set of edge intensities *b^i^*4:  **for** $I\in d_{k}^{i}$** do**5:  Compute *I_g_* by Equation (3) via *I*6:  Compute the enhanced image $I^{\prime }$ via Equation (2) using *I_g_* and *I*7:  **set** I←I′8:  Compute the edge intensity *b* via Equation (5) using *I_g_*. Add it to *b^i^*9:  
**end for**
10:  Compute $\bar{b^{i}}$, the mean of $b^{i}.$ Add it to *B_k_*11:**end for**12:Normalize the mean value of edge intensity $\bar{b^{i}}$ for each category in *B_k_* according to Equation (10) to ${b^{\prime }}_{k}^{i}$13:Compute the quality score *Q^i^* for each category by Equation (11) using ${b^{\prime }}_{k}^{i}.$ Add it to *Q_k_*14:Compute *θ* using *D_k_*, *W_k_*, the embedding vectors during training, and other client prototypes shared by the FedPSFV algorithm15:Compute the margin loss by Equations (12) and (13) using *Q_k_* and *θ*

On this basis, the loss function designed in this paper is shown in Equation (16), where *N* represents the total number of categories in all the client datasets, i.e., the total number of uploaded prototypes; *n* denotes the total number of categories of local clients; *θ_j_* indicates the angle between the feature vector of category *i* and the *j*-th local classifier weight vector; $\varphi_{J}$ represents the angle between feature vector *i* and the *J*-th category center vector of the prototype matrix of all the other clients; *y_i_* denotes the true label index; *m* indicates the margin; and f represents the margin-based loss function mentioned previously.

(16) $$L_{FedPSFV}\;=\;-\log\frac{e^{f{\left(\theta_{y_{i}},m\right)}_{Edge}}}{e^{f{\left(\theta_{y_{i}},m\right)}_{Edge}}\;+\;\sum_{j\;\neq \;y_{i}}^{n}e^{s\cos\theta_{j}}\;+\;\sum_{J\;\neq \;y_{i}}^{N\;-\;n}e^{s\cos\varphi_{J}}}$$

The loss function of the FedPSFV algorithm has not only the comparison term $e^{s\cos\theta_{j}}$, with the local dataset in the denominator, but also the comparison term $e^{s\cos\varphi_{J}}$, with the prototypes of the datasets of the other clients. This feature motivates the model to differentiate between the local embedding vectors and the prototypes of the other clients to increase the feature differentiation ability of the model and compensate for problems such as the slow convergence speed of the individual clients due to the small dataset.

The pseudocode of the client training process and the FedPSFV loss function are shown in Algorithm 2.
**Algorithm 2.** Client training process and FedPSFV loss function**Parameters**: client serial number *k*, model parameter *ω* after the *t*-th round aggregation, and the set of global prototypes $\bar{S}$ after the *t*-th round aggregation**ClientUpdate** (*k*, *ω_t_*, $\bar{S}$):**set** *ω_t_*_,*k*_ ← *ω_t_*1:Compute *W_k_* by Equation (9) via $\bar{S}$ and initialize hyperparameter margins *m*2: 3:**for** each local epoch **do** **for** batch (*x_k_*, *y_k_*)∈*D_k_* **do**4:  Compute loss via Equations (13) and (16) using *x_k_*, *ω_t_*_,*k*_, *W_k_*, and *y_k_*5:  Update local model parameter *ω_t_*_+1,*k*_ according to the loss.6:  **end for**7:**end for**8:Compute local prototype *S_k_* via Equation (6) using *x_k_* and *ω_t_*_+ 1,*k*_9:return *ω_t_*_+1,*k*_, *S_k_*

### 3.4. Global Aggregation

This section introduces the server-side aggregation process, which involves the aggregation of model parameters and the aggregation of prototypes. The server sends the aggregated model parameters and prototype matrix to each client. The entire process is described below.

At the end of each round of communication, the server side receives the updated model parameters of the feature extraction layer from the clients, as well as the prototypes of all the categories of each client. Assuming that the model height of the *k*-th client is represented as *ω_k_*, the server side first aggregates the parameters of the model of the client, as shown in Equation (17),

(17) $$\omega \;=\;\sum_{k\;=\;1}^{m}\frac{\left|D_{k}\right|}{N}\omega_{k}$$

where *ω* denotes the aggregated global model weights and *D_k_* represents the dataset owned by client *k*.

The server then aggregates prototypes from different clients, as shown in Equation (18),

(18) $${\bar{S}}^{\left(j\right)}\;=\;\frac{1}{\left|{CS}_{j}\right|}\sum_{k\in {CS}_{j}}\frac{\left|D_{k}^{j}\right|}{N_{j}}S_{k}^{\left(j\right)}$$

where $\left|D_{k}^{j}\right|$ represents the number of training samples belonging to category *j* in client *k*, *N_j_* denotes the total number of samples of category *j* in the dataset of all the clients, *CS_j_* denotes the set of clients containing category *j*, and $S_{k}^{\left(j\right)}$ denotes the prototype of category *j* uploaded by client *k*. Instead of aggregating multiple prototypes of the same category into a single one, FedPSFV computes the aggregated vector after aggregation and then updates the prototype matrix for each client that owns the category to ensure that the total number of prototypes before and after aggregation remains the same. This process ensures that the shape of the updated weight matrix remains consistent.

The pseudocode for the server-side aggregation algorithm is shown in Algorithm 3.
**Algorithm 3.** Global aggregation**Parameters**: The global prototype set $\left\{\bar{S}\right\}$ excludes the prototypes $\bar{S_{N\;-\;i}}$ owned by client *i*. The server executes:1:Initialize the feature representation layer parameters *ω* of the global model and global prototype set$\;\{\bar{S}\}$2:**for** each round *T* = 1,2……**do**3:  **for** each client *k* in parallel **do**4:  *ω_t_*_+1,*k*_, *S_k_* ← **ClientUpdate** (*k*, *ω*, $\bar{S_{N\;-\;k}}$)5:  
**end for**
6:  Update parameters *ω* of the global model via Equation (17) using *ω_t_*_+1,*k*_7:  Update the global prototype set $\left\{\bar{S}\right\}$ via Equation (18) using *S_k_*8:**end for**

## 4. Experiments

### 4.1. Datasets and Evaluation Methods

In this work, experiments are performed on six public datasets of finger vein images, including SDUMLA-HMT [38], MMCBNU-6000 [39], FV-USM [40], UTFVP [41], VERA [42], and NUPT-FV [43]. The SDUMLA-HMT dataset contains 636 categories, the MMCBNU-6000 dataset contains 600 categories, the FV-USM dataset contains 492 categories, the UTFVP dataset contains 360 categories, the VERA dataset contains 220 categories, and the NUPT-FV dataset contains 1680 categories. Each of the six datasets above contains 6, 10, 6, 4, 2, and 10 images per category, respectively.

In this work, there are six clients, each of which is assigned a public dataset as described above to simulate the process of different organizations collaborating on federated learning training. The background, illumination, and vein clarity of each public dataset vary significantly due to the sampling method. For example, the NUPT-FV dataset was sampled with fingers facing vertically, whereas the other datasets were horizontally oriented. The total number of samples in the VERA dataset was only 440, whereas the total number of samples in the NUPT-FV dataset was 16,800. These differences indicate that the six datasets have different data distributions, which exemplifies the classic non-IID problem in FL [44].

To better evaluate the generalizability of the models trained by the algorithm, this paper uses the open-set testing method from the field of biometrics. We divide the dataset into training and testing sets at a ratio of 8:2 by the number of categories. When federated learning training is performed, only 80% of the categories in each client dataset are involved in training, and the remaining 20% of the categories are not visible to the model. When the performance of the model is tested, all the samples in 20% of the categories are output into embedding vectors via the feature representation layer of the model. Validation pairs are generated via permutations and combinations, and whether they belong to the same category is determined by evaluating whether the similarity of the embedding vectors of the validation pairs is greater than a certain threshold.

In this experiment, the equal error rate (EER) and TAR@FAR = 0.01 were used as evaluation metrics. The equal error rate is the value when the false acceptance rate (FAR) is equal to the false rejection rate (FRR), whereas TAR@FAR = 0.01 is the value of the true acceptance rate (TAR) when the false acceptance rate (FAR) is 0.01. The relevant calculation formulas are shown in (19)–(21),

(19) $$FAR\;=\;\frac{N_{FA}}{N_{IRA}}\;\times \;100\%$$

(20) $$FRR\;=\;\frac{N_{FR}}{N_{GRA}}\;\times \;100\%$$

(21) TAR = 1 − FRR

where *N_IRA_* represents the number of all interclass match pairs, *N_FA_* denotes the number of incorrect acceptances in all interclass match pairs, *N_GRA_* indicates the number of all intraclass match pairs, and *N_FR_* represents the number of incorrect rejections in all intraclass match pairs.

### 4.2. Experimental Results and Analysis

To verify the effectiveness of the FedPSFV algorithm, we conducted four sets of experiments. In the first set of experiments, we compare the FedPSFV algorithm with two cases of client-independent training and client-centralized training to verify the effectiveness of the federated learning framework. In the second set of experiments, the effectiveness of the prototype sharing method in the FedPSFV algorithm and the EdgeAdavein module is verified via ablation experiments. In addition, we compared the performance of the FedPSFV algorithm using different backbone encoders, as well as its robustness under low image contrast conditions. The third set of experiments is compared with existing methods in the field of federated learning and finger vein recognition to verify the superiority of the method in this paper. The fourth set of experiments discusses the communication cost and inference efficiency of the FedPSFV algorithm.

#### 4.2.1. Comparison with Client-Independent Training and Client-Centralized Training Methods

In this experiment, “Local” means that each client is trained independently and that each client does not communicate with the others during training. “Centralized” means that the six datasets are trained together centrally. These two tests simulate the situation under nonfederated learning, and both use the MobileNetV3 model.

The experimental results are shown in Table 1. The table shows that the centralized training method performs better than the local training method; however, the FedPSFV algorithm outperforms both methods. First, the centralized training method does not perform processing, such as image enhancement, and reduces the intraclass distance. Second, FedPSFV increases the interclass distances of different classes in the six datasets by sharing the prototype and the margin-based loss function while decreasing the intraclass distances, thereby increasing the feature extraction ability of the model. In addition, the FedPSFV algorithm is significantly better on VERA and UTFVP, two datasets with small data volumes. Compared with the local and centralized methods, the EER is reduced by 89.15% and 82.82%, respectively, and TAR@FAR = 0.01 is increased by 14.90% and 5.31%, respectively, which indicates that the proposed method can also train a model with high recognition accuracy for clients with small data volumes.

**Figure 4:**
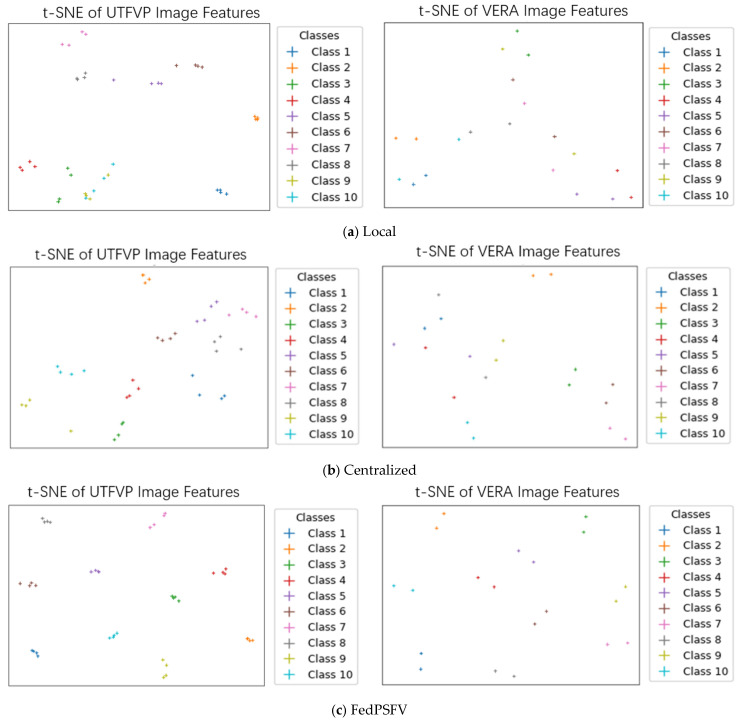
t-SNE images of embedding vectors for the UTFVP and VERA datasets with different training methods.

To further compare the performance of the three methods on small datasets, we also use the t-distributed stochastic neighbor embedding (t-SNE) [45] method to downscale the *d*-dimensional embedding vectors of different instances into 2-dimensional vectors to determine whether the model can indeed increase the interclass distance and decrease the intraclass distance. We randomly selected samples from 10 categories of the UTFVP dataset and the VERA dataset to compute the t-SNE images of their embedding vectors. The results are shown in Figure 4. For the local method, after the extracted embedding vectors are downscaled, the interclass distance of many categories is small, indicating that the model does not distinguish between different categories of samples very well. For the centralized method, the interclass distance is better than that of the Local method because it uses only one model with a larger amount of training data, but the intraclass distance is also larger. For the FedPSFV algorithm, the embedding vectors extracted by its model are able to not only increase the interclass distance well but also reduce the intraclass spacing. These findings prove that FedPSFV is more capable of extracting image features and can increase the distinguishability of sample data.

**Figure 5:**
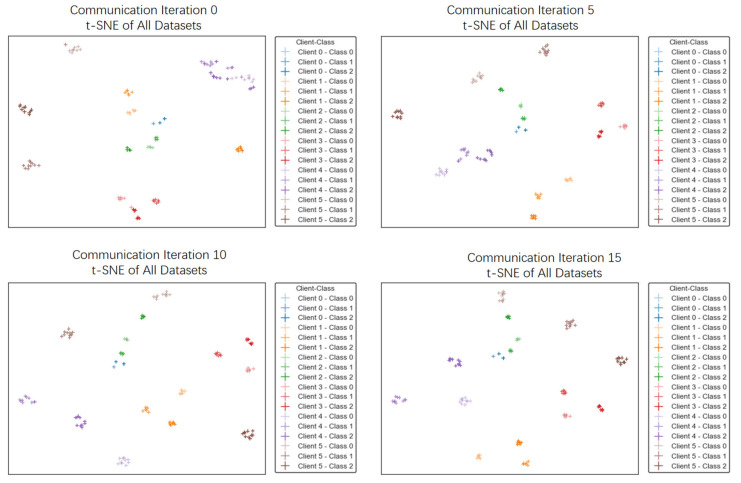
t-SNE image of client embedding vector evolution over communication rounds.

In addition, we visualize the t-SNE plots of embedding vectors from all six client datasets at different communication rounds to verify the effectiveness of FedPSFV for increasing global class separability. Specifically, we select three classes from each client dataset and extract embedding vectors by feeding all 18 classes of data samples into the encoder at communication rounds 0, 5, 10, and 15. The resulting t-SNE visualizations are shown in Figure 5. At the beginning of training, data samples from different clients are combined. However, as training progresses, the interclass distances between data samples from different clients gradually increase.

#### 4.2.2. Ablation Study

In this experiment, first, we conducted ablation studies on the FedPSFV algorithm. Second, we compared the performance of the FedPSFV algorithm when three different backbone encoders were used. Last, we evaluated the robustness of the FedPSFV algorithm under image contrast levels of 0.6, 0.8, and 1.0.

First, the submethod is ablated to verify the effectiveness of the proposed submethod. The FedPSFV algorithm consists of two main submethods: (1) DPS, a method for expanding the classifier weight matrix by sharing prototypes; and (2) *D_AA_*, a method for improving both the image and the image quality indicator of the margin-based loss function. The selection or nonselection of submethods involves the following four methods.

(1) W/O *D_PS_* and *D_AA_* (i.e., baseline method): In the federated learning scenario, MobileNetV3 is used as the basic model, and the margin-based loss function before improvement [37] is used.

(2) W/O *D_AA_*: Based on the baseline method, the classifier weight matrix is expanded by sharing their respective prototypes among different clients and using the aggregated prototypes.

(3) W/O *D_PS_*: Based on the baseline method, image enhancement is used, and the image quality indicator is improved.

(4) FedPSFV: Based on the baseline method, the classifier weight matrix is expanded by sharing the prototypes. The image enhancement and improved image quality indicators are also used.

The experimental results are shown in Table 2. The results of the W/O *D_PS_* and *D_AA_* method is better than those of both the local training method and the centralized training method in Experiment 1, with average EER and TAR@FAR = 0.01 of 1.25% and 97.86%, respectively, which is attributed to the use of the margin-based loss function in the W/O *D_PS_* and *D_AA_* methods. The margin-based loss function can narrow the intraclass distance of the category samples and thereby increase the feature differentiation ability of the model. Compared with the W/O *D_PS_* and *D_AA_* methods, the W/O *D_AA_* and W/O *D_PS_* methods reduce the average EER by 21.60% and 28.80%, respectively, and increase the average TAR@FAR = 0.01 by 1.55% and 0.67%, respectively. This finding indicates that both submethods can increase the feature extraction capability of the model and that the W/O *D_AA_* method provides a greater performance improvement. The FedPSFV algorithm with both submethods achieves the optimal overall performance.

Second, the effect of different encoders on the performance of the FedPSFV algorithm is explored. The main reason for choosing MobileNetV3-Large as the encoder in the FedPSFV framework is its lightweight design, with approximately 3.00 M parameters (excluding the classification layer), making it highly suitable for deployment on mobile devices in finger vein recognition scenarios. To further investigate the effect of the encoder on the performance of FedPSFV, we additionally compare it with two more recent encoders: EfficientNetV2-S [46] and ConvNeXt-Tiny [47]. EfficientNetV2-S contains approximately 24 M parameters, whereas ConvNeXt-Tiny has approximately 29 M parameters. The experimental results are shown in Table 3. The average EERs achieved using EfficientNetV2-S and ConvNeXt-Tiny are 4.19% and 1.69%, respectively. This finding shows that the FedPSFV algorithm has good adaptability to different encoders.

Last, the robustness of the FedPSFV algorithm under low image contrast conditions is discussed. FedPSFV uses gradient-enhanced images, with edge strength serving as an image quality indicator within the classification layer. However, under low-contrast conditions, the gradient-based method may fail to accurately segment vein patterns. To investigate this issue, we manually decreased the image contrast and evaluated the performance of FedPSFV at contrast levels of 0.6, 0.8, and 1.0. As shown in Table 4, lower contrast levels indeed result in performance degradation. For example, on the NUPT dataset, the EER increases from 0.26% to 3.14% and 5.45% at contrast levels of 0.6 and 0.8, respectively, whereas TAR@FAR = 0.01 decreases from 98.92% to 98.84% and 95.12%, respectively. Similar trends can be observed for the VERA dataset. On average, the EER increases from 1.06% to 2.45% and 1.85%, and TAR@FAR = 0.01 decreases from 99.29% to 97.43% and 98.54%, respectively. These results reveal that the FedPSFV algorithm remains robust under low image contrast conditions.

**Figure 6:**
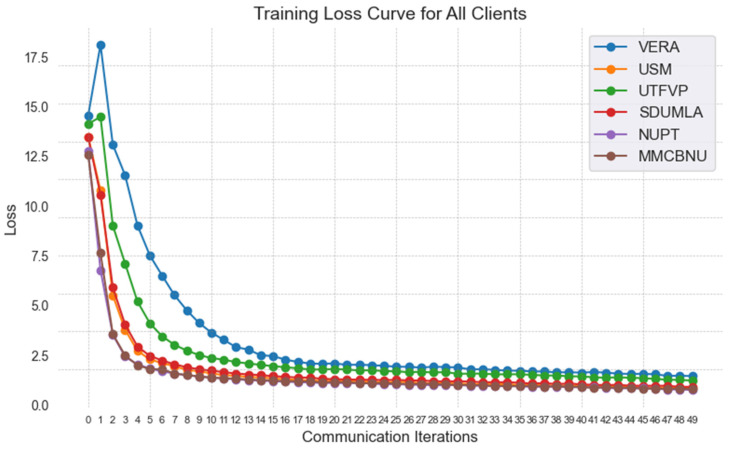
Training loss curves of six clients in the FedPSFV algorithm. All clients are able to converge rapidly.

Figure 6 shows the training loss curve of the FedPSFV algorithm, which contains the training losses of the six clients involved in training. The training loss of the six clients decreases rapidly in the first 15 rounds and then stabilizes gradually, which shows that the FedPSFV algorithm has good generalizability and converges smoothly on six different datasets.

#### 4.2.3. Comparison with Existing Methods

In this experiment, we compare the FedPSFV algorithm with pfedsim [29], FedFV [18], MOON [30], DDP-FV [19], Ditto [10], and FedAS [48]. The results are shown in Table 5, and the FedPSFV algorithm outperforms the comparison methods on the vast majority of datasets, with an average EER of 0.50%, which is 82.82%, 81.48%, 82.27%, 81.95%, 90.22% and 83.97% lower than that of pfedsim, MOON, FedFV, DDP-FV, Ditto, and FedAS, respectively. The average value of TAR@FAR = 0.01 is 99.57%, which is 8.81%, 7.76%, 8.89%, 5.00%, 11.25% and 8.64% higher than those of pfedsim, MOON, FedFV, DDP-FV, Ditto, and FedAS, respectively. For the small dataset UTFVP, the EER of FedPSFV is 75.69–94.54% lower than those of the other methods, and TAR@FAR = 0.01 is 4.58–29.80% higher than those of the other methods. For the small dataset VERA, the EER of FedPSFV is 80.19–88.10% lower than those of the other methods, and TAR@FAR = 0.01 is 10.38–54.80% higher than those of the other methods. These findings prove that FedPSFV can effectively solve the data heterogeneity problem in federated learning because of the difference in data volume and better performance.

#### 4.2.4. Communication Cost and Inference Efficiency

Our FedPSFV achieves superior performance while maintaining manageable communication overhead, as shown in Table 6. The primary contributors to the communication cost are the encoder parameters of MobileNetV3-Large, which account for approximately 3.00 M. Compared with other methods that transmit only model parameters [49,50], FedPSFV introduces additional communication overhead due to prototype sharing. For a prototype dimension of 512, the average upload cost per client increases by only 0.26 M, whereas the download cost increases by 1.56 M, which is acceptable in real-world scenarios where the uplink bandwidth is typically lower than the downlink bandwidth.

Regarding inference time, the primary components of latency in real-time finger vein recognition systems are encoding time and matching time, that is, the time required to process a finger vein image through the encoder to obtain an embedding vector and the time to match this vector against all stored templates in the database. On our mobile-class device, the NVIDIA RTX 4060 laptop (Santa Clara, CA, USA), we evaluated these two components by testing individual samples obtained from all datasets. The encoding time relies on the encoder used; for example, when MobileNetV3-Large is used, the average encoding time is approximately 3.655 ms. The matching time is affected by the number of enrolled identities; for approximately 3980 categories across the six datasets, the average matching time is approximately 1.001 ms. Therefore, the total average inference time is approximately 4.656 ms, which meets the real-time performance requirements of practical finger vein recognition systems.

## 5. Conclusions

In this paper, the finger vein recognition problem in federated learning scenarios is investigated, and the FedPSFV algorithm is designed and implemented. On the basis of the federated learning framework, prototype sharing and margin-based loss functions are presented, which increase the feature discrimination ability of the model and address the performance fluctuation problem caused by differences in datasets. Meanwhile, FedPSFV retains the advantages of a lightweight model and communication efficiency. Comparative experiments show that the FedPSFV algorithm is superior to the existing methods and that the trained model also performs well on clients with small data volumes. For limitations, this study focused exclusively on the application of FedPSFV in federated finger vein recognition. However, FedPSFV is not restricted to this specific domain. The method of improving the loss function by sharing prototypes highlighted in this paper can also be used in general federated learning scenarios, providing a general and efficient solution for model training in heterogeneous data scenarios. Future work will explore the generalizability and effectiveness of FedPSFV in broader federated learning contexts.

## Figures and Tables

**Table 1:** Comparison of local training, centralized training, and FedPSFV.

Datasets	Local		Centralized		FedPSFV	
	EER	TAR@FAR = 0.01	EER	TAR@FAR = 0.01	EER	TAR@FAR = 0.01
SDUMLA	5.15%	89.35%	3.52%	91.84%	0.65%	99.79%
MMCBNU	0.80%	99.31%	0.93%	99.15%	0.08%	100.00%
USM	5.21%	81.31%	4.64%	95.85%	0.40%	99.69%
UTFVP	9.57%	80.71%	4.77%	91.39%	0.53%	99.75%
VERA	6.04%	70.00%	2.60%	90.12%	1.06%	99.29%
NUPT	0.86%	99.26%	1.01%	98.97%	0.26%	98.92%
Average	4.61%	86.66%	2.91%	94.55%	0.50%	99.57%

**Table 2:** Ablation study.

Datasets	W/O *D_PS_* and *D_AA_*		W/O *D_AA_*		W/O *D_PS_*		FedPSFV	
	EER	TAR@FAR = 0.01	EER	TAR@FAR = 0.01	EER	TAR@FAR = 0.01	EER	TAR@FAR = 0.01
SDUMLA	2.71%	95.37%	1.92%	99.25%	2.23%	95.98%	0.65%	99.79%
MMCBNU	0.08%	100.00%	0.39%	99.89%	0.72%	98.94%	0.08%	100.00%
USM	2.64%	95.42%	0.62%	99.45%	0.63%	99.70%	0.40%	99.69%
UTFVP	0.36%	99.98%	0.50%	99.82%	0.29%	100.00%	0.53%	99.75%
VERA	1.36%	98.29%	0.82%	100.00%	1.08%	96.67%	1.06%	99.29%
NUPT	0.33%	98.08%	1.62%	97.85%	0.38%	99.84%	0.26%	98.92%
Average	1.25%	97.86%	0.98%	99.38%	0.89%	98.52%	0.50%	99.57%

**Table 3:** Performance comparison of FedPSFV via different encoders.

Datasets	FedPSFV with Efficientnetv2-S		FedPSFV with ConvNeXt-Tiny		FedPSFV with MobileNetV3 Large	
	EER	TAR@FAR = 0.01	EER	TAR@FAR = 0.01	EER	TAR@FAR = 0.01
SDUMLA	3.60%	91.69%	1.34%	98.61%	0.65%	99.79%
MMCBNU	1.35%	98.20%	0.84%	99.49%	0.08%	100.00%
USM	0.47%	99.83%	0.31%	99.90%	0.40%	99.69%
UTFVP	5.38%	84.32%	0.48%	99.60%	0.53%	99.75%
VERA	13.57%	60.00%	6.62%	85.52%	1.06%	99.29%
NUPT	0.79%	99.29%	0.54%	99.45%	0.26%	98.92%
Average	4.19%	88.89%	1.69%	97.10%	0.50%	99.57%

**Table 4:** Performance of FedPSFV at different image contrast levels.

Datasets	Contrast 0.6		Contrast 0.8		Contrast 1.0	
	EER	TAR@FAR = 0.01	EER	TAR@FAR = 0.01	EER	TAR@FAR = 0.01
SDUMLA	0.56%	99.79%	0.92%	99.18%	0.65%	99.79%
MMCBNU	0.04%	99.98%	0.28%	99.95%	0.08%	100.00%
USM	0.40%	99.87%	0.44%	99.87%	0.40%	99.69%
UTFVP	0.80%	99.60%	0.21%	100.0%	0.53%	99.75%
VERA	2.45%	97.43%	1.85%	98.54%	1.06%	99.29%
NUPT	3.14%	98.84%	5.45%	95.12%	0.26%	98.92%
Average	1.23%	99.25%	1.53%	98.78%	0.50%	99.57%

**Table 5:** Performance of FedPSFV and the comparison methods.

Datasets	pfedsim		MOON		FedFV		DDP-FV		Ditto		FedAS		FedPSFV	
	EER	TAR@FAR = 0.01	EER	TAR@FAR = 0.01	EER	TAR@FAR = 0.01	EER	TAR@FAR = 0.01	EER	TAR@FAR = 0.01	EER	TAR@FAR = 0.01	EER	TAR@FAR = 0.01
SDUMLA	2.20%	96.33%	3.31%	93.05%	2.64%	94.93%	2.52%	96.23%	6.21%	90.19%	2.92%	95.34%	0.65%	99.79%
MMCBNU	0.45%	99.78%	0.79%	99.42%	0.95%	99.04%	0.61%	99.63%	0.94%	99.12%	1.08%	98.82%	0.08%	100.00%
USM	2.65%	94.64%	1.71%	96.77%	2.19%	95.17%	3.24%	90.26%	4.36%	88.96%	3.62%	89.04%	0.40%	99.69%
UTFVP	2.18%	95.38%	2.92%	92.36%	3.20%	91.27%	2.90%	93.81%	9.71%	76.85%	4.81%	86.61%	0.53%	99.75%
VERA	8.91%	64.14%	6.61%	73.60%	7.04%	69.05%	6.40%	89.95%	8.33%	83.12%	5.35%	80.90%	1.06%	99.29%
NUPT	1.09%	98.79%	0.83%	99.17%	0.89%	99.19%	0.92%	99.12%	1.13%	98.75%	0.92%	99.19%	0.26%	98.92%
Average	2.91%	91.51%	2.70%	92.40%	2.82%	91.44%	2.77%	94.83%	5.11%	89.50%	3.12%	91.65%	0.50%	99.57%

**Table 6:** Per-round upload and download overhead using the MobileNetV3 encoder.

	Upload	Download
pfedsim, MOON, FedFV, DDP-FV, Ditto, FedAS	3.00 M	3.00 M
FedPSFV	3.00 M + 0.26 M	3.00 M + 1.56 M

## Data Availability

The data presented in this study are publicly available in the SDUMLA-HMT [38], MMCBNU-6000 [39], FV-USM [40], UTFVP [41], VERA [42], and NUPT-FV [43] datasets.
